# Supplementary material for: Alterations in Cerebellar Microtubule Cytoskeletal Network in a ValproicAcid-Induced Rat Model of Autism Spectrum Disorders
Source: Biomedicines. 2022 Nov 24;10(12):3031. doi: 10.3390/biomedicines10123031 (PMC9776106; doi:10.3390/biomedicines10123031)
Supplement: Supplementary file 1 [file biomedicines-10-03031-s001.zip › biomedicines-1924302-supplementary final revised.pdf]

Supplementary Materials:

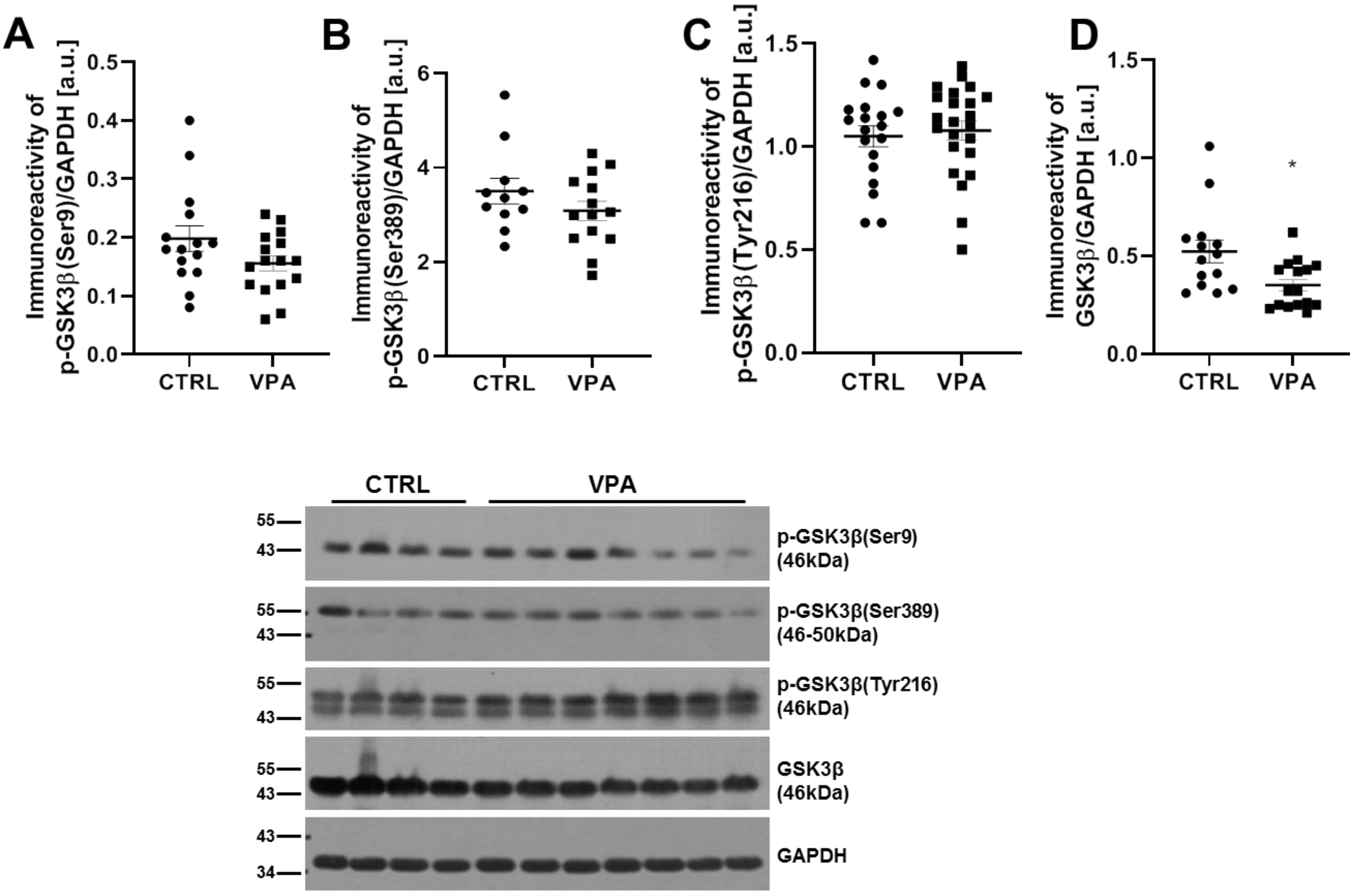

Supplementary Figure S1. The effect of prenatal exposure to VPA on the GSK-3β in the cerebellum of adolescent rat offspring. The phosphorylation status of GSK-3β at (Ser9), (Ser389) and (Tyr216) as well as the immunoreactivity of total GSK-3β in control and VPA-exposed rats were monitored using Western blot analysis. Densitometric analysis and representative pictures of pGSK-3β(Ser9) **(A)**, pGSK-3β(Ser389) **(B)**, pGSK-3β(Tyr216) **(C)** and total GSK-3β **(D)** in the cerebellum are shown. Results were normalised to GAPDH levels. Data represent the means ± S.E.M. from n=(10-16) independent experiments. * *p<0.5*, *** *p<0.001*, vs. control.

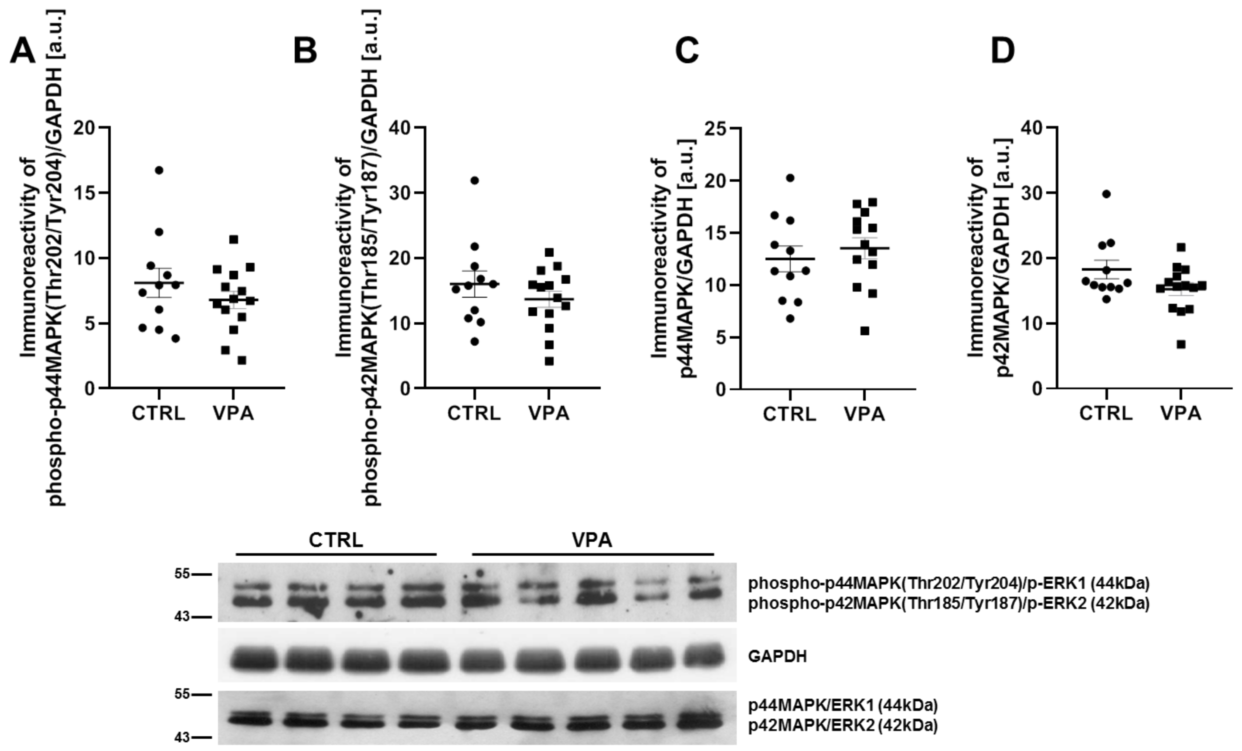

Supplementary Figure S2. The effect of prenatal exposure to VPA on the p44/p42MAPK (ERK1/2) in the cerebellum of adolescent rat offspring. Immunoreactivity of phospho-ERK1(Thr202/Tyr204), phospho-ERK2(Thr185/Tyr187), ERK1 and ERK2 were monitored using Western blot analysis. Densitometric analysis and representative pictures of phospho-ERK1, phospho-ERK2 **(A, B)** as well as total ERK1 and ERK2 **(C, D)** in the cerebellum are shown. Results were normalised to GAPDH levels. Data represent the means ± S.E.M. from n=(11-14) independent experiments.

Supplementary Results

GSK-3β activity was evaluated by measurement of the phosphorylation status at (Ser9) and (Ser389), which are molecular marks of deactivation of GSK-3β as well as (Tyr216), associated with an increase in GSK-3β activity. Moreover, its protein level was evaluated. As presented in Figure 1, the immunoreactivity of p-GSK-3β(Ser9) remained unchanged (**Figure 1A**), just like p-GSK-3β(Ser389) levels (**Figure 1B**) in the cerebellum of VPA offspring. Also, no changes in the level of p-GSK-3β, phosphorylated at (Tyr216) were observed in the VPA animals, compared to control (**Figure 1C**). In addition, the level of GSK-3β was significantly decreased (by about 33%, *p=0.0121*) in the cerebellum of VPA-exposed animals (**Figure 1D**). All the above data indicate no effect of VPA exposure on GSK-3β activity in the cerebellum of the offspring. Thus, the data indicate GSK-3β-independent Tau hyperphosphorylation induced by VPA.

To study the possible involvement of mitogen-activated protein kinases: ERK1 and ERK2 in VPA-evoked Tau phosphorylation, we analysed the level of phosphorylated p44MAPK (p-ERK1) at (Thr202/Tyr204), p42MAPK (p-ERK2) at (Thr185/Tyr187) as well as the level of total p44/42MAPK (ERK1/2). Our study revealed that exposure to VPA during embryonic development had no effect on the level of p-ERK1 and p-ERK2 in the cerebellum (**Figure 2A** and **B**). Analysis of the expression of total ERK1/2 also revealed the lack of changes in the protein level of ERK1/2 in this brain structure of animals exposed to VPA (**Figure 2C** and **D**). The data indicate ERK1/2-independent Tau hyperphosphorylation evoked by VPA administration.

Table S1. Experimental conditions used to perform the Western blot experiments.

Primary antibody	Brand/cat #	Dilution
Rabbit anti- α/β-tubulin	Cell Signalling<br>#2148S	1:1000<br>5% BSA in TBS-T 0.1%
Mouse anti- Tau	Santa Cruz Biotechnology<br>sc-32274	1:500<br>5% milk in TBS-T 0.1%
Mouse anti- pTau(Ser396)	Cell Signalling<br>#9632	1:250<br>TBS-T 0.1%
Rabbit anti- pTau(Ser199/202)	Sigma-Aldrich<br>T6819	1:1000<br>5% milk in TBS-T 0.1%
Rabbit anti- pTau(Ser416)	Cell Signalling<br>#15013P	1:1000<br>5% milk in TBS-T 0.1%
Mouse anti- pGSK-3β(Ser9)	Santa Cruz Biotechnology<br>sc-373800	1:250<br>5% milk in TBS-T 0.1%
anti- pGSK-3β(Ser389)	Proteintech<br>14850-1-AP	1:500<br>5% milk in TBS-T 0.1%
Mouse anti- pGSK-3β(Tyr216)	BD Diagnostic<br>612313	1:250<br>0.1% BSA in TBS-T 0.1%
Rabbit anti- GSK-3β	Cell Signalling<br>#9315	1:1000<br>5% milk in TBS-T 0.1%
Mouse anti- pp44/pp42MAPK(Thr202/Tyr204)	Cell Signalling<br>#9106	1:1000<br>TBS-T 0.1%
Mouse anti- p44/p42MAPK	Cell Signalling<br>#4696	1:1000<br>5% milk in TBS-T 0.1%
Rabbit anti- p35/p25	Cell Signalling<br>#2680 and<br>Santa Cruz Biotechnology<br>sc-820 (Mix)	1:1000<br>1% BSA in TBS-T 0.1%
Mouse anti- αII-spectrin	Santa Cruz<br>Biotechnology<br>sc-46696	1:1000<br>5% milk in TBS-T 0.1%
Rabbit anti- p-AMPK(Thr172)	Cell Signalling<br>#50081	1:500<br>TBS-T 0.1%
Rabbit anti- AMPK	Cell Signalling<br>#5831	1:500<br>TBS-T 0.1%
Mouse anti- MAP1B	Santa Cruz<br>Biotechnology<br>Sc-365668	1:500<br>1% BSA in TBS-T 0.1%
Rabbit anti- MAP2	Cell Signalling<br>#8707S	1:1000<br>5% BSA in TBS-T 0.1%
Rabbit anti- p-MAP2(Ser136)	Cell Signalling<br>#4541S	1:1000<br>5% BSA in TBS-T 0.1%
Mouse anti- MAP6 (STOP)	Santa Cruz<br>Biotechnology<br>sc-137036	1:250<br>5% milk in TBS-T 0.1%
Mouse anti- NF-L	Santa Cruz<br>Biotechnology<br>sc-20012	1:125<br>5% milk in TBS-T 0.1%
Rabbit anti- GAPDH	Sigma-Aldrich<br>G9545-200UL	1:50000<br>5% milk in TBS-T 0.1%
Rabbit anti- vinculin	Cell Signalling<br>#13901	1:1000<br>5% milk in TBS-T 0.1%
**Secondary antibody**	**Brand/cat #**	**Dilution**
anti-mouse IgG	GE Healthcare<br>VXA931V	1:4000<br>5% milk in TBS-T 0.1%

anti-rabbit IgG	Sigma-Aldrich<br>A0545-1ML	1:8000<br>5% milk in TBS-T 0.1%
